# Decision support during electronic prescription to stem antibiotic overuse for acute respiratory infections: a long-term, quasi-experimental study

**DOI:** 10.1186/s12879-017-2602-7

**Published:** 2017-07-31

**Authors:** Jeneen Gifford, Elisabeth Vaeth, Katherine Richards, Tariq Siddiqui, Christine Gill, Lucy Wilson, Sylvain DeLisle

**Affiliations:** 1 Veterans Affairs Maryland Health Care System, Baltimore, MD USA; 2 School of Medicine, University of Maryland, Baltimore, MD USA; 3 Maryland Department of Health and Mental Hygiene, Baltimore, MD USA; 4 School of Pharmacy, University of Maryland, Baltimore, MD USA; 5 Professor of Medicine and Clinical Sciences, University of Texas Southwestern, 8B, Building 2, Dallas VA Medical Center, 4500 S Lancaster Rd, Dallas, TX 75216 USA

## Abstract

**Background:**

Interventions to support decision-making can reduce inappropriate antibiotic use for acute respiratory infections (ARI), but they may not be sustainable. The objective of the study is to evaluate the long-term effectiveness of a clinical decision-support system (CDSS) interposed at the time of electronic (e-) prescriptions for selected antibiotics.

**Methods:**

This is a retrospective, observational intervention study, conducted within a large, statewide Veterans Affairs health system. Participants are outpatients with an initial visit for ARI. A CDSS was deployed upon e-prescription of selected antibiotics during the study period. From 01/2004 to 05/2006 (pre-withdrawal period), the CDSS targeted azithromycin and the fluoroquinolone gatifloxacin. From 05/2006 to 12/2011 (post-withdrawal period), the CDSS was retained for azithromycin but withdrawn for the fluoroquinolone. A manual record review was conducted to determine concordance of antibiotic prescription with ARI treatment guidelines.

**Results:**

Of 1131 included ARI visits, 380 (33.6%) were guideline-concordant. For azithromycin, concordance did not change between the pre- and post-withdrawal periods, and adjusted odds of concordance was 8.8 for the full study period, compared to unrestricted antibiotics. For fluoroquinolones, guideline concordance decreased from 88.6% (39 of 44 visits) to 51.3% (59 of 115 visits), pre- vs. post-withdrawal periods (*p* < 0.005). The adjusted odds of concordance compared to “All Other Antibiotics” visits decreased from 24.4 (95% CI 9.0–66.3) pre-withdrawal to 5.5 (95% CI 3.5–8.8) post-withdrawal (*p* = .008). Concordance did not change between those same time periods for antibiotics that were never subjected to the intervention (“All Other Antibiotics”).

**Conclusions:**

A CDSS interposed at the time of e-prescription of selected antibiotics can shift their use toward ARI treatment guidelines, and this effect can be maintained over the long term as long as the CDSS remains in place. Removal of the CDSS after 3.5 years of implementation resulted in a rise in guideline-discordant antibiotic use.

**Electronic supplementary material:**

The online version of this article (doi:10.1186/s12879-017-2602-7) contains supplementary material, which is available to authorized users.

## Background

Antibiotic overuse promotes the emergence of resistant microorganisms [1] and contributes to preventable morbidity and costs [2]. In outpatient settings, most antibiotic prescriptions are directed at acute respiratory infections (ARI) [3]. Misutilization of antibiotics for uncomplicated ARI remains common, years after publication of widely endorsed prescribing guidelines [4]. The rationale for the persistence of the problem is not fully understood, and may include lack of knowledge of the treatment guidelines, [5] lack of appreciation for the development of resistance, [6] and pressure to expediently satisfy perceived patient expectations [6]. Provider education can decrease needless antibiotic prescriptions for ARI, but no information delivery method has proven consistently effective, scalable and sustainable [7, 8].

The broad deployment of electronic medical records (EMR) is opening new educational avenues to medical providers. EMR-based clinical decision support systems (CDSS) can be integrated into the workflow and change clinical practice by providing timely, guideline-based, patient-specific recommendations [9]. A CDSS with a light footprint could conceivably remain in place indefinitely [10]. However, our knowledge on how to assure that such a tool remains useful over the long-term is still maturing.

In an effort to improve outpatient antibiotic stewardship at a large, statewide Veterans Affairs health system, a CDSS was interposed at the time of electronic order entry for two antibiotics commonly used for ARI: gatifloxacin, the mainstream formulary respiratory fluoroquinolone when it was available, and azithromycin. This intervention nearly eliminated unwarranted ARI prescriptions for the two agents for a period of at least 3.5 years [11]. When gatifloxacin was withdrawn from the market in May 2006 due to reports of dysglycemias [12], we considered the possibility that, after 3.5 years, the guideline information delivered by the CDSS with each attempt to prescribe an antibiotic had become assimilated into our standard of practice, thereby rendering the CDSS unnecessary. To gain insight into this possibility, we did not reapply the ARI CDSS to moxifloxacin, the formulary replacement for gatifloxacin. Meanwhile, the CDSS for azithromycin was left in place for a period of more than 4.5 years. In the current work, we take advantage of these conditions to simultaneously ask if the CDSS retained its effectiveness over the long-term and if its effect on decreasing antibiotic overuse for ARI could persist despite withdrawing the intervention.

## Methods

This is a retrospective, observational study designed to assess the long-term effects of CDSS use and the consequence of CDSS withdrawal on concordance of antibiotic usage with widely-endorsed ARI treatment guidelines. The study was performed using medical record data from the Veterans Affairs Maryland Health Care System (VAMHCS) during a period extending from January 1, 2004, to December 31, 2011. The ARI CDSS was prompted each time an attempt was made to electronically prescribe azithromycin for the entire study period, or gatifloxacin from the beginning of the study period until May 2006 (pre-withdrawal period), when gatifloxacin was removed from the market. The CDSS did not target other antibiotics, including moxifloxacin which replaced gatifloxacin on the formulary after May 2006 (post-withdrawal period). Only one oral formulation of a respiratory fluoroquinolone was listed on the national VA formulary at any given time, with minimal overlap of listing during transitions. No non-formulary respiratory fluoroquinolones, such as levofloxacin, were prescribed for any of the ARI visits included in our study sample.

### Description of the intervention

The ARI CDSS has been previously described [11]. For targeted antibiotics, the CDSS was deployed when electronic (e-) prescription was attempted. For azithromycin and gatifloxacin, the CDSS included treatment paths for the following diseases: community-acquired pneumonia, acute bronchitis, acute sinusitis, non-specific acute respiratory infection (ARI), and exacerbations of chronic obstructive pulmonary disease (COPD). An “Other” option was included for provider-supplied rationales. The CDSS used data mined from the EMR and information entered by the provider to assess whether an antibiotic prescription would be consistent with published ARI guidelines. Diagnostic criteria and antibiotic prescribing criteria have been previously defined [11]. For appropriate rationales, the CDSS led to order entry for the antibiotic and generated documentation describing why the drug was being used. If antibiotics could be safely withheld, the CDSS did not lead to a prescription. Instead, it provided guidance on how to maintain patient satisfaction when withholding antibiotics. For both safety and tolerability, providers could override the system by providing their own rationale in the “Other” pathway or by prescribing antibiotics not subject to the CDSS.

### Study participants

An automated case-detection algorithm (CDA) previously found to identify 73% of patients with ARI was applied to EMR-derived relational databases [13]. The algorithm flagged outpatient visits if providers assigned an ARI-related diagnostic code OR prescribed a cough suppressant AND if the clinical notes documented at least two ARI symptoms, as assessed by automated text analysis [13].

### Record review

A record review was completed for 1) all flagged visits associated with azithromycin, gatifloxacin, or moxifloxacin prescription, 2) a random sample of all flagged visits associated with “All Other Antibiotics”, limited to 50% of flagged visits to retain feasibility of the manual record review, and 3) a random sample of 200 flagged visits where no antibiotics were given, as such visits had rarely been found to be discordant in the past [11].

Sampled flagged visits were manually abstracted for data elements needed to assign diagnosis and treatment for pneumonia, acute bronchitis, or sinusitis [11]. Three independent reviewers, including a pulmonary medicine specialist, examined all free-text EMR entries on the day of index for the flagged visits, with the exception of notes issued by the CDSS itself. Presence or absence of individual ARI symptoms and related time courses were abstracted. A stated diagnosis of pneumonia or inclusion of pneumonia in the list of differential diagnoses was coded as pneumonia. Chest imaging was not reviewed. Reviewers cross-validated 10% of each other’s work. Inter-rater reliability was determined for: a) the application of inclusion/exclusion criteria (kappa = 81.0%); and b) for the final assessment of concordance of antibiotic prescribing with guidelines (kappa = 85.5%).

### Exclusion, diagnostic and concordance criteria

After the automated CDA identified possible ARI visits, pre-determined exclusion criteria were applied using manually abstracted information. These criteria included: 1) Stated diagnosis of COPD; 2) Did not meet case definition for outpatient sinusitis, acute bronchitis, or pneumonia; 3) Not an initial visit for a given ARI episode; 4) No provider documentation; 5) Antibiotic unequivocally prescribed for a non-ARI diagnosis; 6) Records could not be accessed for review. Cases of pharyngitis were excluded from our study according to criteria 2 because it was not an available pathway for the CDSS and because the CDSS-targeted antibiotics were found not to be misused in patients whose only ARI diagnosis was pharyngitis [11].

Abstracted information for each visit was reassembled into ARI diagnoses and visit concordance was determined according to the previously described case definitions and treatment guidelines [11]. Except for pneumonia, ARI diagnoses were not mutually exclusive. A visit was considered “concordant” if antibiotic use followed guidelines for at least one ARI diagnosis.

### Antibiotic usage

The Veterans Affairs Pharmacy Benefits Management Services database was queried to compare national to VAMHCS usage of azithromycin and fluoroquinolones (ProClarity software, Microsoft Corp, Redmond CA).

### Statistical analysis

Based on the accuracy of the screening case-detection algorithm [13] and allowing for attrition due to misclassification, we calculated that abstracting 1800 flagged outpatient encounters would result in at least an 80% power to detect a 10% change in concordance after CDSS withdrawal.

The potential confounders for the relationship between study time periods and guideline concordance were identified using T-tests for continuous variables and Chi-square tests for categorical variables. Patient-specific covariates were sex, self-reported race, age, and documented ARI symptoms. Covariates were included in the multivariable logistic regression if they were found to have an interaction, according to standard practice. Multivariable logistic regression and odds ratios were calculated to determine the association between CDSS withdrawal and guideline concordant antibiotic prescribing (SAS software v. 9.2, SAS Institute Inc., Cary, NC). A chi-square test was used to compare guideline concordance between pre-withdrawal and post-withdrawal periods. A *p*-value of .05 or less was considered statistically significant.

## Results

### Study population

**Fig. 1:**
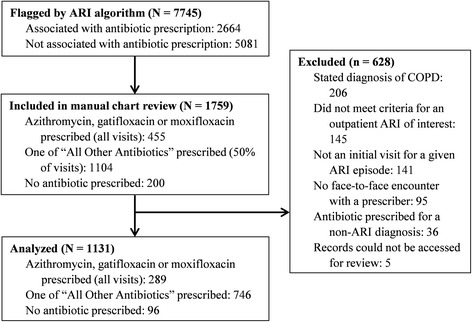
Study flow diagram. The population was primarily male (82.5%) and older. Cough was the most common ARI symptom, followed by sputum production and fever/chills/night sweats. ARI symptoms were similar whether or not antibiotic usage was concordant with guidelines (Table 1)

Over the 8 year study period, the ARI CDA flagged 7745 visits. A total of 1759 visits were reviewed, including all visits during which azithromycin, gatifloxacin, or moxifloxacin was prescribed (*n* = 455). Of the 1759 visits manually reviewed, 628 were excluded according to the pre-defined criteria. The study included 1131 visits for 1118 patients with an initial visit for ARI. These results are summarized in Fig. 1.

### Concordance by diagnosis

Of the 1131 total ARI visits, 380 (33.6%) were guideline concordant, including 285 visits that resulted in an antibiotic prescription for ARI, as well as 95 of the 96 ARI visits where no antibiotics were given. Providers diagnosed pneumonia in 227 visits (20% of all ARI visits). All pneumonia patients received antibiotics, which constituted the majority of the study’s concordant visits (*n* = 227/380 or 59.7%). Of the 751 discordant ARI visits, 750 (99.9%) visits were discordant because antibiotics were prescribed when they were not warranted. In only one visit were antibiotics not prescribed when the guidelines suggested that they should be considered, a patient with acute sinusitis who had experienced sinus pain and/or tenderness and purulent nasal drainage for 7 or more days. Acute bronchitis was the sole diagnosis in 467 (62.2%) discordant visits, and sinusitis was the sole diagnosis in 31 (4.1%) discordant visits; 252 discordant visits (33.6%) met criteria for both acute bronchitis and sinusitis (Table 1).

**Table 1:** Characteristics of study visits. Characteristics include patient demographics, symptoms present during the index visit, and ARI conditions for which diagnostic criteria were met

Characteristics	Overall (*n* = 1131)	Concordant Antibiotic Visits^a^ (*n* = 380)^b^	Discordant Antibiotic Visits (*n* = 751)^c^
Sex, n (%)			
Male	933 (82.5)	340 (89.5)	593 (79.0)
Female	198 (17.5)	40 (10.5)	158 (21.0)
Self-Reported Race, n (%)			
African American	559 (49.4)	219 (57.6)	340 (45.3)
White	489 (43.2)	142 (37.4)	347 (46.2)
Other/Missing	83 (7.3)	19 (5.0)	64 (8.5)
Age at Encounter Date, years			
Mean	55.1	56.7	54.3
Median	54.0	55.0	54.0
Range	20.0–93.0	23.0–90.0	20.0–93.0
Symptoms			
Cough, new or changed in past 21 days	1076 (95.1)	356 (93.7)	720 (95.9)
Fevers, chills, or night sweats	541 (47.8)	212 (55.8)	329 (43.8)
Facial pain or tenderness	302 (26.7)	90 (23.7)	212 (28.2)
Purulent nasal drainage	221 (19.5)	80 (21.1)	141 (18.8)
Sinus symptoms for >7 days	114 (10.1)	61 (16.1)	53 (7.1)
New or worsening sputum	698 (61.7)	215 (56.6)	483 (64.3)
Unilateral sinus symptoms	39 (3.5)	17 (4.5)	22 (2.9)
Worsening after initial improvement	13 (1.2)	5 (1.3)	8 (1.1)
ARI Condition^d^			
Pneumonia, all	227 (20.0)	227 (59.7)	0 (0.0)
Pneumonia only	198 (17.5)	198 (52.1)	0 (0.0)
Sinusitis, all	392 (34.7)	108 (28.4)	284 (37.8)
Sinusitis only	44 (3.9)	13 (3.4)	31 (4.1)
Acute bronchitis, all	860 (76.0)	140 (36.8)	720 (95.9)
Acute bronchitis only	541 (47.8)	74 (19.5)	467 (62.2)

^a^Concordant antibiotic visits include both: a) visits for which antibiotics were warranted and were prescribed, and b) visits for which antibiotics were not warranted and were not prescribed. For the ARI condition “Acute bronchitis only”, all 74 visits were concordant because no antibiotics were prescribed

^b^Percentages for this column are calculated with Concordant Antibiotic Visits (*n* = 380) as the denominator

^c^Percentages for this column are calculated with Discordant Antibiotic Visits (*n* = 751) as the denominator

^d^ARI conditions are not mutually exclusive, so the column percentages for this section will not total 100%

### CDSS exposure is associated with guideline concordance

The CDSS was applied in 130 visits leading to azithromycin prescriptions during the whole study period, 81 (62.3%) of which were concordant, and in 44 visits leading to fluoroquinolone (gatifloxacin) prescriptions during the pre-withdrawal period, 39 (88.6%) of which were concordant (Table 2). By comparison, when visits resulted in the prescription of antibiotics that were never subjected to the CDSS during the study period (“All Other Antibiotics”), 106 of 746 (14.2%) were concordant. The adjusted odds of concordance were 8.8 (95% CI 5.7–13.6) for ARI visits leading to azithromycin prescriptions and 24.4 (95% CI 9.0–66.3) for ARI visits leading to gatifloxacin prescriptions when compared to visits for which “All Other Antibiotics” were prescribed. The CDSS was discontinued for 115 visits leading to fluoroquinolone prescriptions during the post-withdrawal period, and adjusted odds of guideline concordance was 5.5 times higher (95% CI 3.5–8.8) than for all visits from the group “All Other Antibiotics”.

**Table 2:** Odds of guideline concordant prescription for ARI and CDSS exposure

Antibiotic Prescribed During ARI Visit	No. of visits (n, %)^a^	Guideline Concordant (n, %)^b^	Unadjusted, odds ratio (95% CI)^c^	Adjusted for age, sex, race, and symptoms (Logistic Regression model), odds ratio (95% CI)^c^
Azithromycin^d^	130 (11.5)	81 (62.3)	9.5 (6.3–14.3)	8.8 (5.7–13.6)
Fluoroquinolone, pre-withdrawal	44 (3.9)	39 (88.6)	36.5 (13.8–96.7)	24.4 (9.0–66.3)
Fluoroquinolone, post-withdrawal	115 (10.2)	59 (51.3)	7.5 (4.8–11.8)	5.5 (3.5–8.8)
All Other Antibiotics^d^	746 (66.0)	106 (14.2)		
No Antibiotics	96 (8.5)	95 (99.0)		

^a^Number of visits for which the antibiotic is prescribed / Total number of visits (*n* = 1131)

^b^Number of concordant visits for which the antibiotic is prescribed / Number of total visits for which the antibiotic is prescribed

^c^Versus “All Other Antibiotics”

^d^For the groups “Azithromycin” and “All Other Antibiotics”, data are shown for the entire 8 year study period

### CDSS withdrawal decreases concordance

The CDSS targeted the fluoroquinolone gatifloxacin during the 3.5 year pre-withdrawal period. The CDSS was not implemented for the fluoroquinolone moxifloxacin for the 4.5 year post-withdrawal period. For those fluoroquinolones, guideline concordance decreased from 88.6% (39 of 44 visits) during the pre-withdrawal period to 51.3% (59 of 115 visits) post-withdrawal (*p* = .002). The corresponding adjusted odds of concordance compared to “All Other Antibiotics” visits decreased from 24.4 (95% CI 9.0–66.3) pre-withdrawal to 5.5 (95% CI 3.5–8.8) post-withdrawal (*p* = .008). For azithromycin, the CDSS remained in place for the entire 8 year study period, and there was no significant difference in guideline concordance between the pre-withdrawal period (49/69 or 71.0%) and the post-withdrawal period (32/61 or 52.5%). There was also no significant difference in concordance for the other control group, “All Other Antibiotics”, between the pre-withdrawal period (44/268 or 16.4%) and the post-withdrawal period (62/478 or 13.0%).

### CDSS withdrawal increases fluoroquinolone use

**Fig. 2:**
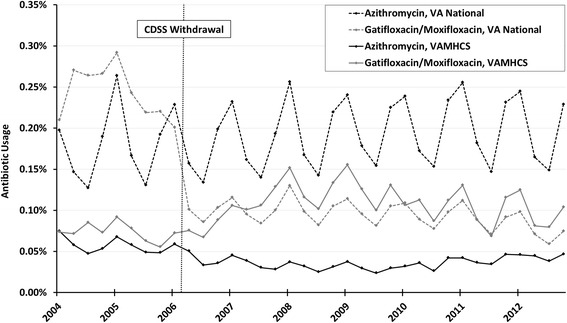
Comparison of national and Maryland VA usage of CDSS targeted antibiotics. The ratio (Number of prescriptions / All drug prescriptions) is multiplied by 100 and the percentage is reported on the y-axis. The *vertical dotted line* indicates when moxifloxacin replaces gatifloxacin in the VA formulary and defines the pre- and post-withdrawal periods. Note the marked seasonality of usage for azithromycin and the fluoroquinolones

VA pharmacy antibiotic usage is reported as a ratio comparing the total number of prescriptions for a single antibiotic and the total number of prescriptions for all drugs per quarter. During the pre-withdrawal period, VAMHCS usage was lower than national VA usage for both azithromycin (Fig. 2, solid black versus dashed black lines) and gatifloxacin (solid gray versus dashed gray lines). In the post-withdrawal period, average use of azithromycin remained lower at VAMHCS than nationwide (solid vs. dashed black line). Nationally, fluoroquinolone use decreased from the pre- to the post-withdrawal period (dashed gray line). Within VAMHCS, the trend was in the opposite direction, with usage of moxifloxacin rising (solid gray line) to ultimately match national usage.

Out of 381 visits during the pre-withdrawal period, 39 (10.2%) were associated with guideline concordant fluoroquinolone use, and 5 (1.3%) were associated with non-guideline concordant fluoroquinolone use. Had this relative utilization been maintained with the 654 visits in the post-withdrawal period, we would have expected 67 (10.2% of 654) visits with guideline concordant and 9 (1.3% of 654) visits with non-guideline concordant fluoroquinolone prescriptions. In actuality, there were fewer than expected guideline concordant prescriptions (59 out of 654 or 9.0%) and much more than expected guideline discordant prescriptions (56 out of 654 or 8.6%). These data suggest that the observed increase in fluoroquinolone usage shown in Fig. 2 could be accounted for by an increase in discordant prescriptions.

## Discussion and conclusions

We studied the long-term effect of a CDSS interposed at the time of e-prescription on concordance with ARI treatment guidelines. The sustained effectiveness of the CDSS was demonstrated in two ways. First, over an implementation period of 3.5 years (for gatifloxacin) to 8 years (for azithromycin), the proportion of concordant visits was higher with the CDSS than without. Second, concordance decreased when the CDSS was withdrawn for the fluoroquinolone, without a corresponding change for antibiotics either continuously (azithromycin) or never subjected to the intervention. Together, these data indicate that a CDSS at the time of prescription can be an effective, long-term strategy for curtailing misuse of the drugs that it targets. This effect may not persist after the CDSS is removed, but CDSS can be designed to be a durable part of provider workflow.

We took several steps to strengthen this study’s quasi-experimental design: a) to minimize sampling bias, we obtained a population-based sample of ARI visits through a previously validated ARI CDA; b) we used a manual reference review and explicit criteria to identify ARI diagnoses and concordance with treatment guidelines; c) we compared the change in guideline concordance when the CDSS was withdrawn against two control groups, one continuously exposed to the CDSS during the whole study period (azithromycin), and one never exposed to the intervention (“All Other Antibiotics”); d) we did not implement complementary educational interventions, thereby increasing the likelihood that the observed effects on prescribing practices could be attributed to the CDSS itself; and e) we compared VAMHCS to national drug utilization to show that the local increase in fluoroquinolone use following CDSS withdrawal ran contrary to national trends, and thus was unlikely to simply be the result of perceived advantages of moxifloxacin over gatifloxacin. Our findings of stable azithromycin use and increased fluoroquinolone use during the post-withdrawal period also ran contrary to those observed in a national study of one million VA ARI visits, where azithromycin usage increased and fluoroquinolone usage decreased in 2012 compared to 2005 [14].

The sustainability of the CDSS intervention distinguishes it from other attempts to address antibiotic stewardship for ARI. Financial incentives, clinician workshops, or audits with providers can all decrease unnecessary antibiotics for ARI [15–19]. Because these interventions are resource intensive, they usually cannot be continued indefinitely. The durability of their effect therefore depends upon changing the antibiotic prescribing culture. Of the three published examples of outpatient interventions whose effect was maintained for multiple years, two featured voluntary participation and may thus have selected for particularly motivated providers [20, 21]. Another was conducted within small, stable practices, i.e. in environments expected to be amenable to cultural changes [22]. Our results suggest that it is possible to foster the judicious use of antibiotics in health care settings that experience a continuous influx of new trainees and part-time staff, i.e. where it may be difficult to provide timely information about prescribing guidelines through conventional means [16]. Our results suggests that the effectiveness of the CDSS required its continued implementation. This is nevertheless possible because as the CDSS becomes an integral part of the workflow and providers become familiar with its informational content, it ultimately consumes minimal resources,

We do not know what propelled the rate of discordant antibiotic prescriptions once the CDSS was discontinued. Some providers may not have assimilated the ARI treatment guidelines, either because of insufficient CDSS exposures, or because they limited their interaction with the CDSS to what was strictly needed to generate a prescription thus missing the scientific arguments that would lead to a permanent change in behavior. Other providers may have internalized the content of the CDSS, but this knowledge may not have been sufficient to maintain concordant practices in the presence of external pressures such as time expediency, patient or subspecialist demands, or ongoing performance measures of patient satisfaction [23]. Given these realities, the process-of-care into which the CDSS operated included non-informational elements that may indeed prove crucial to antibiotic management, such as providing implicit institutional support for the decision to withhold antibiotics, or requiring providers to commit a written justification for the use of antibiotics to the EMR, where it is potentially vulnerable to subsequent peer review.

Our study has some limitations that warrant consideration. The biases associated with a pre−/post- intervention study design have been described [24]. The intervention was deployed in a veteran population that is mostly male, is older, and has more co-morbidities than the general population [25]. Providers within the VA system have long used the EMR for virtually all aspects of patient care, and this familiarity may have an impact on the acceptance and effectiveness of the CDSS. We did not formally reassess the performance characteristics of the ARI CDA. However, the CDA yielded a validated ARI case density commensurate with past performance measurements. Moreover, the structured components of the algorithm (cough suppressant prescriptions, ARI diagnostic codes) are not expected to be susceptible to changes in language patterns. In the unlikely possibility that the free-text describing common respiratory symptoms changed over the years, there is no reason to believe that these changes would systematically create a bias for or against concordance for any specific antibiotic group. The retrospective data collection meant that symptoms and signs were assumed to be absent if they were not documented in the medical record. ARI episodes could therefore have been missed or mislabeled, along with the rationale justifying the use of antibiotics. Our study focused mostly on ARI visits for which antibiotics were prescribed, and so could not assess possible shunting of utilization between different antibiotics. However, a prior assessment that included all ARI visits indicated that the CDSS intervention did not transfer inappropriate use from the CDSS-targeted agents to alternative antibiotics, nor did it promote the diagnosis of pneumonia to justify prescribing antibiotics [11]. If we excluded visits where the antibiotic was clearly prescribed for a non-ARI diagnosis, we did not evaluate how frequently providers supplied rationales other than those provided by ARI guidelines to prescribe CDSS-targeted agents. We limited CDSS targeting to azithromycin and fluoroquinolones, because they were the two most commonly prescribed antibiotics for ARI and were not commonly used for diseases other than ARI [11]. Different effect sizes may therefore have been observed if different or additional antibiotics had been targeted, or if the CDSS was implemented in health systems with different prescribing patterns [14, 16]. Our study did not collect information about emergence of resistant organisms, clinical efficacy or patient safety. However, no patient with provider-assigned diagnosis of pneumonia failed to receive antibiotics, a finding consistent with our past work with 2125 patients [11]. Other studies have demonstrated no increases in patient follow-up visits, need for antibiotic prescription after the index visit, or mortality in cohorts where antibiotic stewardship programs successfully decreased over-prescribing for ARI [26–29]. Taken together, these limitations represent design opportunities for future studies aimed at extending e-prescription-based approaches to other antibiotics, drugs, diseases or settings.

There is a continued need for better methods to promote responsible antibiotic use, and any intervention must match the scale and historical resilience of the problem. A CDSS systematically interposed at the time of e-prescription provided significant and durable improvements in the use of antibiotics for outpatients with ARI. The approach could be adapted to optimize the use of any medication and take advantage of the increasing integration of the EMR in medical practices.

## Additional files

Additional file 1: Coded dataset for the study. (XLS 263 kb)

Additional file 2: Legend for supplementary data file. (PDF 155 kb)

## Abbreviations

ARI: Acute respiratory infection
CDA: Case-detection algorithm
CDSS: Clinical decision-support system
CI: Confidence interval
COPD: Chronic obstructive pulmonary disease
EMR: Electronic medical record
VAMHCS: Veterans Affairs Maryland Health Care System
