# Supplementary material for: Decision support during electronic prescription to stem antibiotic overuse for acute respiratory infections: a long-term, quasi-experimental study
Source: BMC Infect Dis. 2017 Jul 31;17:528. doi: 10.1186/s12879-017-2602-7 (PMC5537944; doi:10.1186/s12879-017-2602-7)
Supplement: Supplementary file 2 — Legend for supplementary data file. (PDF 155 kb) [file 12879_2017_2602_MOESM2_ESM.pdf]

Table 3. Legend for supplementary data file.

Table Heading	Description	
PatID	Unique code for individuals with visits included in the study	
EpisodeNumber	1 = First visit for an individual<br>2 = Second visit for an individual	
LUTName	Antibiotic prescription associated with visit, according to pharmacy records<br>1= Azithromycin<br>2= Gatifloxacin<br>3= Moxifloxacin<br>4= Penicillin V<br>5= Other<br>Blank= None prescribed	
PrescriptionMade	1= “LUTName” is 1, 2, 3, 4, or 5<br>2= “LUTName” is 0	
PurulentNasalDrainage	Purulent nasal drainage (thick or discolored drainage)	1= Symptom present<br><br>0= Symptom not present
PainTenderness	Maxillofacial sinus and/or tooth pain/tenderness (including reported frontal headache)	
SinusSxs7days	Sinusitis-related symptom duration of greater than or equal to 7 days	
Unilateral	Unilateral sinus-related symptoms (pain, tenderness or swelling)	
Worseningafterimprovement	Worsening of sinus-related symptoms after initial improvement	
Feverchillsnightsweats	New fever (subjective or objective[≥ 37.8C/100F]), chills or night sweats within the last 7 days	
Sorethroat	Sore throat	
Cough21	New cough of duration < 21 days	
Cough7	New or changed cough within last 7 days	
Sputum	New or changed sputum production within last 7 days	
Dyspnea	New or changed dyspnea within last 7 days	
Pleuriticchestpain	New or changed pleuritic chest pain within last 7 days	
AntibioticPrescribed	1= Visit documentation indicates that an antibiotic was prescribed<br>0= Visit documentation does not indicate that an antibiotic was prescribed	
AbxforOtherDx	1= Antibiotic explicitly prescribed for a non-ARI diagnosis<br>0= Antibiotic not explicitly prescribed for a non-ARI diagnosis	
AcutePneumonia	1= Visit meets criteria for pneumonia<br>2= Visit does not meet criteria for pneumonia	
AcuteSinusitis	1= Visit meets criteria for acute sinusitis<br>2= Visit does not meet criteria for acute sinusitis	
AcuteBronchitis	1= Visit meets criteria for acute bronchitis<br>2= Visit does not meet criteria for acute bronchitis	
ARIofInterest	1= Symptoms lead to a diagnosis of interest (pneumonia, sinusitis, bronchitis)<br>0= Symptoms do not lead to a diagnosis of interest	
Appropriateness	1= Antibiotic prescription or non-prescription was appropriate based on the ARI treatment guidelines<br>0= Antibiotic prescription or non-prescription was not appropriate	

	based on the ARI treatment guidelines
PrePost	1= Visit occurred before the date of change from gatifloxacin to moxifloxacin<br>0= Visit occurred after the date of change from gatifloxacin to moxifloxacin
Race	Patient race, self-reported. Options are:<br>White<br>Black or African American<br>Asian<br>Native Hawaiian or other Pacific Islander<br>American Indian or Alaskan Native<br>Unknown by patient<br>Declined to answer
